# *Mentha rotundifolia* (L.) Huds. aqueous extract attenuates H_2_O_2_ induced oxidative stress and neurotoxicity

**DOI:** 10.3389/fnins.2023.1121029

**Published:** 2023-03-09

**Authors:** Khadija Boualam, Nezha Bouhaddou, Mansour Sobeh, Mohamed Tabyaoui, Khalid Taghzouti

**Affiliations:** ^1^Physiology and Physiopathology Team, Genomics of Human Pathologies Research Center, Faculty of Sciences, Mohammed V University in Rabat, Rabat, Morocco; ^2^Materials, Nanotechnology and Environment Laboratory LMNE, Faculty of Sciences, Mohammed V University in Rabat, Rabat, Morocco; ^3^AgroBioSciences, Mohammed VI Polytechnic University, Ben Guerir, Morocco

**Keywords:** *Mentha rotundifolia* (L.) Huds, oxidative stress, neurotoxicity, behavioral impairments, polyphenols

## Abstract

**Introduction:**

Oxidative stress plays a causal role in neurodegenerative diseases. The aim of this study is to evaluate the antioxidant and neuroprotective effects of *Mentha rotundifolia* (L.) Huds (*M. rotundifolia*), a widely used Moroccan plant in traditional medicine.

**Methods:**

The chemical composition of *M. rotundifolia* aqueous extract was analyzed by liquid chromatography coupled to mass spectrometry (LC-MS). 2,2-diphenyl 1-picrylhydrazyl (DPPH) and 2,2′-azino-bis 3-ethylbenzothiazoline-6-sulfonic acid (ABTS^+^) assays were used to assess its *in vitro* antioxidant activity. H_2_O_2_ was utilized to induce oxidative stress and neurotoxicity *in vivo*. Behavioral changes were evaluated using Open Field, Y-maze and Rotarod tests. Hyperalgesia was assessed using the tail immersion test.

**Results and discussion:**

The LC-MS/MS analysis revealed high content of kaempferol glucuronide (85%) at the extract. IC_50_ values of the DPPH and ABTS were 26.47 and 41.21 μg/mL, respectively. Pre-treatments with *M. rotundifolia* extract attenuated the behavioral changes induced by H_2_O_2_. In addition, the latency of tail withdrawal increased significantly in the treated groups suggesting central analgesic effect of *M. rotundifolia* extract. Moreover, the extract attenuated the deleterious effects of H_2_O_2_ and improved all liver biomarkers. The obtained results suggested that *M. rotundifolia* had remarkable antioxidant and neuroprotective effects and may prevent oxidative stress related disorders.

## 1. Introduction

The mitochondrial electron transport chain plays a key role in the cell’s energy production *via* a series of electron transfer reactions (Rich and Maréchal, 2010). Under physiological conditions, up to 4% of electrons may escape and react directly with dissolved oxygen in the cytoplasm, giving rise to reactive oxygen species (ROS). The latter are either free radicals such as superoxide anion (O_2_**^.–^**) or hydroxyl radical (OH**^.–^**), molecules such as hydrogen peroxide (H_2_O_2_), or singlet oxygen (^1^O_2_) (Haleng et al., 2007). Although they are involved in several complex physiological processes such as growth regulation, apoptosis, blood pressure regulation, cognitive function, and immune defense (Brieger et al., 2012), their elevated levels generate a state of oxidative stress, which plays a major role in the occurrence of chronic and degenerative diseases, among them neurotoxicity (Pham-Huy et al., 2008).

The body has several defense mechanisms to control the cellular ROS levels to avoid getting into a status of oxidative stress. These include endogenous antioxidants, as a first defense line, such as catalase, superoxide dismutase, and glutathione peroxidase. Antioxidants are the second defense line comprising ascorbic acid, uric acid, glutathione, α-tocopherol, and ubiquinol and the third defense line includes DNA repair enzyme systems and proteolytic enzymes (Ighodaro and Akinloye, 2018). However, in case of elevated ROS levels, a supply of exogenous antioxidants, such as natural plant extracts, polyphenols, vitamins, is crucial to reduce ROS dependent damage.

The genus *Mentha*, a member of the mint family, Lamiaceae, contains around 42 species with a sub-cosmopolitan distribution across all agroclimatic conditions. This genus is widely known for its essential oils that are valued at over US$ 400 million (Bounatirou et al., 2007). Different preparations (Tinctures, decoction, and tablets) of the genus are traditionally used to treat several ailments, such as bad breath, gingivitis and ondotalgies, menstrual cramps, respiratory and gastrointestinal infections. These preparations are also used as sedative, diuretic, carminative and antispasmodic agents (El Hassani, 2020).

The Cuban mint, *Mentha rotundifolia* (L.) Huds. is a hybrid perennial herb derived from a cross between *M. suaveolens* and *M. longifolia* (Denslow and Poindexter, 2009). This plant is traditionally used as a remedy for pain with antiseptic and anti-inflammatory agents to treat wounds and infections (Riahi et al., 2019). Furthermore, it owns antioxidant, anti-tyrosinase, anti-acetylcholinesterase and insecticidal activities (Fatiha et al., 2015; Ben Haj Yahia et al., 2019). These properties are mainly attributed to the presence of several phenolic compounds with rosmarinic acid as a dominant metabolite (Siham et al., 2019).

Several antioxidant therapeutic approaches are currently being investigated for the treatment of oxidative stress-related pathologies, among them neurological diseases. Some of which are currently undergoing clinical trials (Forman and Zhang, 2021). In fact, treatment with free radical scavengers or antioxidants to enhance the antioxidant defenses or decrease the pro-oxidant production may be effective in preventing, treating, or even stopping many neurological diseases (Teleanu et al., 2019). In this context, we aim to study, *in vivo*, the antioxidant effect of *M. rotundifolia* leaves aqueous extract. We here explored the antioxidant properties of *M. rotundifolia* leaves aqueous extract *in vitro* using ABTS and DPPH assays and *in vivo* against H_2_O_2_-induced neurotoxicity and oxidative stress. Behavioral changes and hepatic biomarkers (AST, ALT, and bilirubin) were also assayed. Finally, we characterized the phytoconstituents of the aqueous extract of *M. rotundifolia* using liquid chromatography coupled with mass spectroscopy technique (LC-MS/MS).

## 2. Materials and methods

### 2.1. Plant material and extraction

*Mentha rotundifolia* (L.) Huds. leaves were collected at the Mkam Tolba village, located near Khemisset city (33°55′41.9″ N, 6°16′34.2″ W), Morocco, during the flowering period (August 2022). The leaves were dried in shade for 2 weeks at room temperature. The dried leaves were ground, and a 100 g was infused in hot distilled water (1000 mL × 90°C) for 15 min. The infusion was then filtered, reduced under vacuum, and lyophilized yielding a crude extract (13.43 ± 1.04%, *n* = 3).

### 2.2. Chemical composition characterization

A Shimadzu system coupled to an MS 8050 triple quadrupole mass spectrometer was utilized. Separation was performed using a C18 reversed phase column (Zorbax Eclipse XDB-C18, 4.6 mm × 150 mm, 3.5 μm, Agilent, USA). A gradient of water and acetonitrile (ACN) (0.1% formic acid each) was applied from 5 to 30% ACN over 45 min and then increased to 58% ACN for the next 15 min with a flow rate of 1 mL/min. The sample was automatically injected using an autosampler SIL-40C XS autosampler. The instrument was controlled by LabSolutions software. Ions were detected in the negative ion mode, a full scan mode and a mass range of 100–1500 m/z (Tawfeek et al., 2023).

### 2.3. *In vitro* antioxidant activity

#### 2.3.1. DPPH assay

The free radical scavenging activity of *M. rotundifolia* aqueous extract was tested using 2,2-diphenyl-1-picryl-hydrazyl (DPPH.), taking ascorbic acid as reference standard. A volume of 0.5 mL of DPPH (0.2 mM) was added to the extract at different concentrations ranging from 10 to 100 μg/mL. The reaction was then incubated for 30 min in the dark at room temperature (Boualam et al., 2021). The absorbance was read at 517 nm, and the scavenging activity was estimated using the following equation line:


$$\mathrm{Free}\mathrm{radical}\mathrm{scavenging}\left(\%\right)=\frac{\left(O.D\,\mathrm{control}-O.D\,\mathrm{test}\right)}{\left(O.D\,\mathrm{control}\right)}*100$$

The experiment was performed three times.

#### 2.3.2. ABTS assay

The test is based on the ability of an antioxidant to stabilize the blue-green colored cationic radical ABTS**.**^+^ (2,2- azinobis-3-ethylbenzothiazoline-6-sulfonate) by trapping a proton and transforming it into colorless ABTS^+^. ABTS^+^ solution (1.9 mL) has been added to 600 μL of extract solution at different concentrations (10–1000 μg/mL). After incubation for 7 min at room temperature, the absorbance was measured at 734 nm (Boualam et al., 2021). The blank was prepared by replacing the extract solution with methanol. The scavenging activity was estimated using the following equation:


$$\mathrm{Free}\mathrm{radical}\mathrm{scavenging}\left(\%\right)=\frac{\left(O.D\,\mathrm{control}-O.D\,\mathrm{test}\right)}{\left(O.D\,\mathrm{control}\right)}*100$$

The experiment was performed three times.

### 2.4. *In vivo* antioxidant activity

#### 2.4.1. Animals and experimental design

The experiments were performed on Wistar rats weighing 180–200 g and raised at the central animal care facilities of the Faculty of Sciences, Mohammed V University of Rabat, Morocco. Animals were randomly housed in polyethylene cages with a free access to food and water in a room with controlled temperature (22 ± 1°C) and under a 12 h light–dark cycle. The animals were divided randomly to the following groups with their respective treatment:

- Group 1 and 2 received 1 mL saline intraperitoneally (i.p) for 21 days.

- Group 3 and 4 received 62.5 mg/kg *M. rotundifolia* extract (i.p) for 21 days.

- Group 5 and 6 received 125 mg/kg *M. rotundifolia* extract (i.p) for 21 days.

- Group 7 and 8 received 250 mg/kg *M. rotundifolia* extract (i.p) for 21 days.

**FIGURE 1:**
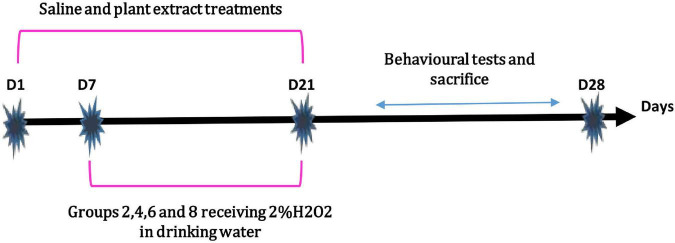
Experimental design of the *in vivo* study.

At day 8, 2% H_2_O_2_ was added to the drinking water of groups 2, 4, 6, and 8, for the remaining 15 days of treatment as described at Figure 1 (Bakour et al., 2018; Bouayed and Soulimani, 2019). During the treatment period, the animals were weighed every 5 days to highlight the effect of H_2_O_2_ on the body weight (BW) and to evaluate the potential protective effect of *M. rotundifolia* aqueous extract against H_2_O_2_ toxicity.

#### 2.4.2. Behavioral tests

##### 2.4.2.1. Open field test

The open field test (OF) is a common measure of exploratory behavior and general locomotor activity in mice and rats (Gould et al., 2009). It’s also used to assess the animal’s anxiety behavior through parameters such as the number of defecations or areas visited (Seibenhener and Wooten, 2015). The OF device used in this study is a square black device with a surface area of 80 cm^2^, with a height of 45 cm. The ground of the field is divided with white strips into 25 squares of 15 cm × 15 cm each, Supplementary Figure 1. This test is performed in a dimly lit room where the animal is placed at the center. The field is cleaned after each test. Each animal spent 10 min in the OF. Locomotor activity is evaluated by the distance covered by the animal (the number of tiles crossed) and the number of sit-ups performed. Anxiety-Like symptoms were assessed by the number of entries in the central zone of the OF and the time spent in this zone.

##### 2.4.2.2. Rotarod test

The rotarod test is widely used to evaluate the motor coordination in rodents (Shiotsuki et al., 2010). The apparatus is made of a plastic rotating cylinder, 7 cm in diameter and 28 cm long, fixed around a horizontal axis raised by 30 cm and divided into 4 compartments of 9 cm width each, Supplementary Figure 2 (Panlab, Harvard Apparatus). The test is performed in an isolated room with normal lighting and programmed over two phases: The pre-test phase where the animals undergo habituation and the test phase where the rats are placed on the rotating cylinder at a fixed speed of 4 rotations per min (rpm). Once the animal is stable, the apparatus is adjusted to increase the speed of rotation until a maximum speed of 40 rpm is reached, which is maintained fixed until the end of the test. The time spent on the cylinder does not exceed 180 s.

##### 2.4.2.3. Y-maze test

The Y-maze test can be used to assess both spatial working and spatial reference memories in rodents (Kraeuter et al., 2019). It’s a black Y-shaped device with three arms orientated at 120° angles from each other, Supplementary Figure 3. For the spatial working memory, the spontaneous alternation is calculated. For this, the number of arm entries and alternations (consecutive entries into all three arms) are recorded to calculate the percentage of the alternation behavior defined by the following formula (Kraeuter et al., 2019):


%⁢Alternation=(Number⁢of⁢Alternations/[Total⁢number⁢of⁢arm⁢entries-2])⁢×⁢ 100

As for reference spatial memory, it is organized in two phases: the training session, when the animal is placed in the Y-maze device with one arm closed off and determined as the novel arm. This session is followed by the test session, where the animal is placed back in the device after a 1 h time interval. The animal should remember which arm was not explored previously and should visit this novel arm more frequently than the others. The number of entries into the novel arm is then compared to the entries into the other arms to assess the degree of spatial memory.

##### 2.4.2.4. Tail immersion test for hyperalgesia

Hyperalgesia is defined as a state of enhanced intensity of pain sensation induced by either noxious or ordinarily non-noxious stimulation of peripheral tissues (Taïwe and Kuete, 2017). Hyperalgesia was assessed using tail immersion test described by Janssen et al. (1963). Briefly, the animals were placed in the restraint cylinder, and the tail tip was submerged in a water bath at 50 ± 0.5°C. A flick of the tail away from the heat source is produced as a nociceptive reflex is spinally mediated (Bannon and Malmberg, 2007). The latency of the tail withdrawal reflex was measured. Each immersion was terminated after 15 s to minimize potential skin damage.

### 2.5. Determination of hepatic parameters

All animals were sacrificed by decapitation 24 h after the last test and overnight fasting. Blood samples were collected, and the serum was separated for assaying the liver biomarkers. The serum levels of ALT, AST and total bilirubin were assessed according to the standard methods described in Garber (1981) and Huang et al. (2006).

### 2.6. Statistical analysis

Data were analyzed using GraphPad Prism 8. Normality was assessed by the Shapiro-Wilk test. IC_50_ values were presented as means ± SD (*n* = 3) and were compared using *t*-test. *In vivo* test results were analyzed using two-ways ANOVA considering the treatment and exposure to H_2_O_2_ as variation factors. Bonferroni’s multiple comparisons test was used whenever there was significance. Results are expressed as means ± SD (*n* = 6). The differences were regarded as statistically significant at *p* ≤ 0.05.

## 3. Results

### 3.1. Chemical composition

**FIGURE 2:**
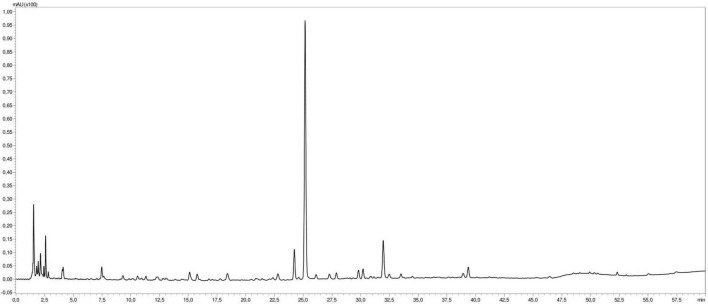
Liquid chromatography profile of *Mentha rotundifolia* leaves aqueous extract.

Liquid chromatography analysis of *M. rotundifolia* leaves aqueous extract revealed forty phytoconstituents mainly phenolic acids, flavonoids, and their glucosides (Table 1 and Figure 2). Kaempferol glucuronide dominated the extract with a relative abundance of 85%.

**TABLE 1:** Annotated phytoconstituents from *Mentha rotundifolia* leaves aqueous extract using LC-MS/MS.

Rt (min)	(M-H)^–^	MS/MS	Compound names
1.12	191	111	Quinic acid
1.87	133	115	Malic acid
4.63	463	331, 169	Galloyl glucose pentose
4.93	315	153	Dihydroxybenzoic acid glucoside
5.11	359	197	Syringic acid glucoside
5.53	339	165	Quinyl phloretic acid
6.97	447	315, 153	Dihydroxybenzoic acid glucosyl pentoside
7.28	353	191	Chlorogenic acid
7.52	315	153	Dihydroxybenzoic acid glucoside
8.42	447	315, 153	Dihydroxybenzoic acid glucosyl pentoside
9.08	137	108	Hydroxybenzoic acid
9.38	299	137	Hydroxybenzoic acid glucoside
9.92	349	165	Syringyl phloretic acid
10.04	337	163	Coumaroylquinic acid
10.16	295	163	Coumaric acid pentoside
10.76	341	179	Caffeoyl glucose
11.12	253	121	Benzoic acid pentoside
12.19	353	191	Chlorogenic acid isomer
12.50	265	147	Phenylacetyl cinnamic acid
13.53	305	179	Gallocatechin
14.01	337	163	Coumaroylquinic acid
15.21	341	179	Caffeoyl glucose
16.73	593	383, 353	Apigenin C-diglucoside
16.83	355	193	Feruloyl glucose
17.74	367	193	Feruloyl quinic acid
19.30	335	173	Caffeoyl shikimic acid
22.72	289	245	(epi)Catechin
23.20	623	447, 285	Kaempferol glucosyl glucuronide
24.39	593	285	Kaempferol rutinoside
24.95	447	285	Kaempferol glucoside
25.29	461	285	Kaempferol glucuronide
29.22	359	197	Rosmarinic acid
29.28	431	269	Apigenin glucoside
29.34	447	285	Kaempferol glucoside
29.70	445	269	Apigenin glucuronide
31.08	359	161	Rosmarinic acid isomer
32.99	461	285	Kaempferol glucuronide
39.44	493	359	Salvianolic acid
42.42	537	197	Salvianolic acid I
45.57	269	151	Apigenin

### 3.2. *In vitro* antioxidant activity

*Mentha rotundifolia* aqueous extract furnished solid antioxidant activities in DPPH and ABTS assays (IC_50_ ≤ 50 μg/ml) in a concentration-dependent manner (Table 2 and Supplementary Figures 4A, B). The obtained results might be attributed to the high contents of polyphenolic compounds, among them phenolic acids and flavonoids.

**TABLE 2:** IC_50_ values of *Mentha rotundifolia* aqueous extract *via* DPPH and ABTS assays.

Assay	DPPH	ABTS
	IC_50_ value (μg/mL)	
Extract	26.47 ± 1.68**	41.21 ± 2.54****
Ascorbic acid	1.74 ± 0.28	–
Trolox	–	3.48 ± 2.49

Results were presented as mean ± SD. Comparison was made to the corresponding reference compounds using t-test.

**p ≤ 0.01.

****p ≤ 0.0001.

### 3.3. *In vivo* results

#### 3.3.1. Body weight monitoring

**FIGURE 3:**
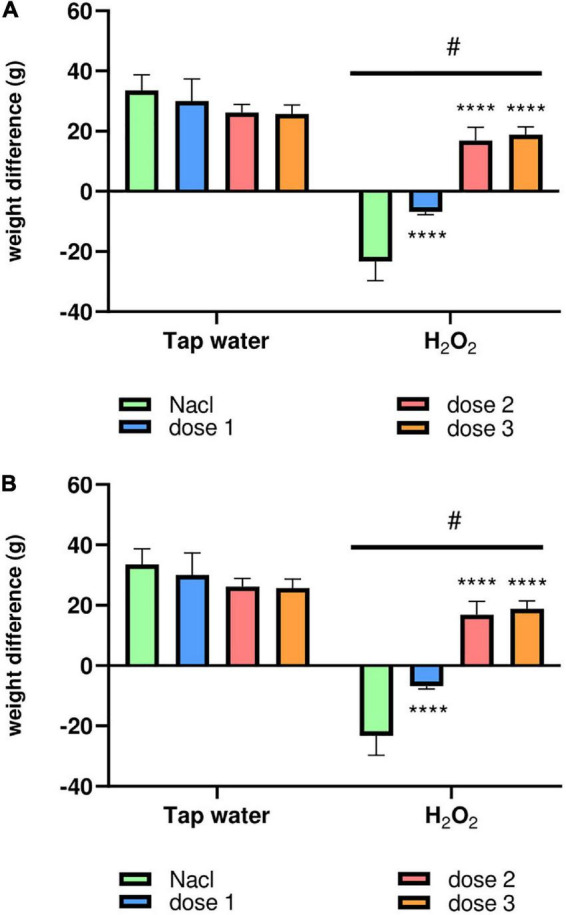
The animals’ body weight variation during the test period. **(A)** The kinetics of the average body weight of the different groups of animals weighed every 5 days of the treatment period. **(B)** The average body weight intake of all the animal groups between the first and the last day of treatment. The results were presented as mean ± SD (*n* = 6). Data were analyzed by Two-ways ANOVA followed by Bonferroni’s *post-hoc* test; ****p ≤ 0.0001 compared to saline group in the same treatment conditions. (#) in comparison with the control group (group 1).

Animals were weighed each 5 days during the test period to highlight the effect of oxidative stress on the body weight (BW) and to evaluate the potential protective effect of *M. rotundifolia* aqueous extract against H_2_O_2_-induced toxicity. As awaited, H_2_O_2_ intake significantly decreased the body weight of animals (Group 2) compared to the control group (Group 1) starting from the day 15th of the treatment, Figures 3A, B. Interestingly, *M. rotundifolia* extract at two tested doses (125 and 250 mg/kg) attenuated these effects starting from the 10th day of treatment. Noteworthy, there was no significant differences between the treated groups with the extract only (Groups 3, 5, and 7) and the control group (Group 1) at the end of the experiment, Figures 3A, B.

#### 3.3.2. Behavioral tests

##### 3.3.2.1. Open field test

###### 3.3.2.1.1. Locomotion

**FIGURE 4:**
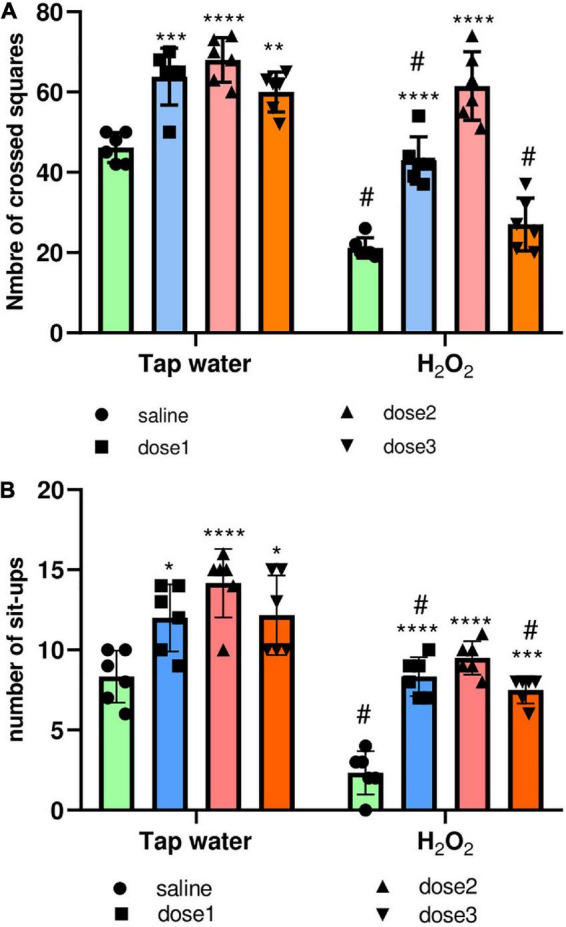
Effect of the interaction between the extract treatment and the exposure to H_2_O_2_ on locomotion assessed by the OF test. **(A)** The distance crossed by animals defined by the number of squares. **(B)** The number of animals’ sit-ups. The results were presented as mean ± SD (*n* = 6). Data were analyzed by Two-ways ANOVA followed by Bonferroni’s *post-hoc* test; *p ≤ 0.05, **p ≤ 0.01, ***p ≤ 0.001, ****p ≤ 0.0001 compared to saline group in the same treatment conditions. (#) in comparison with the control group (group 1).

The locomotion of animals was assessed using the OF test. For this, the total distance crossed by the animal and the number of sit-ups (when the animal stands on its two hind paws) were calculated. As expected, H_2_O_2_ intake remarkably reduced the number of crossed squares of animals (Group 2) compared to control group (Group 1), *p* < 0.0001, Figure 4A. *M. rotundifolia* extract at the two tested doses (62.5 and 125 mg/kg) reversed the effects of H_2_O_2_, Figure 4A. Again, H_2_O_2_ intake notably declined the number of sit-ups (number of times the animals stood on their hind paws) of animals (Group 2) compared to control group (Group 1), *p* < 0.0001, Figure 4B. Noticeably, *M. rotundifolia* extract at the three tested doses (62.5, 125, and 250 mg/kg) ameliorated the effects of H_2_O_2_ with no significant differences between the three doses, Figure 4B. Noteworthy to highlight, the numbers of crossed squares and sit-ups were significantly increased in the extract treated groups (*p* ≤ 0.05) in normal rats (Tap water), Figures 4 A, B.

##### 3.3.2.2. Anxiety-like behavior

**FIGURE 5:**
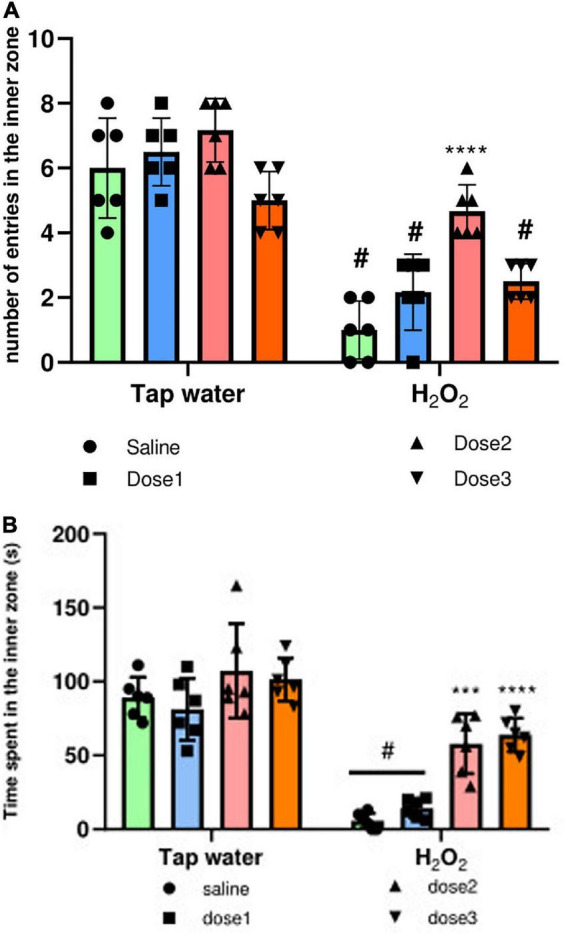
Effect of the interaction between the extract treatment and the exposure to H_2_O_2_ on Anxiety-like symptoms assessed by the OF test. **(A)** The number of entries in the inner zone of the OF. **(B)** The time spent in the inner zone. The results were presented as mean ± SD (*n* = 6). Data were analyzed by Two-ways ANOVA followed by Bonferroni’s *post-hoc* test; ***p ≤ 0.001, ****p ≤ 0.0001 compared to saline group in the same treatment conditions. (#) in comparison with the control group (group 1).

The anxiety-like behavior of animals was evaluated using the OF test, where the number of entries and the time spent in the inner zone were calculated. Agreeing with the above assays, H_2_O_2_ intake significantly (*p* ≤ 0.0001) decreased the number of entries to the inner zone of the groups 2, 4, and 8, Figure 5A. These effects were inverted in 125 mg/kg *M. rotundifolia* extract treated group which has a comparable number of entries than control group (group 1), Figure 5A. Again, H_2_O_2_ intake significantly decreased the time spent in the inner zone compared to the control group (Group 1), Figure 5B. Animals treated with *M. rotundifolia* (125 and 250 mg/kg doses) spent significantly more time in the inner zone compared to H_2_O_2_ group (Group 2) and their times were comparable to the control group (Group 1), Figure 5B. There were no significant differences between the extract treated groups (Groups 3, 5, and 7) and control group (Group 1) for both parameters.

##### 3.3.2.3. Rotarod test

**FIGURE 6:**
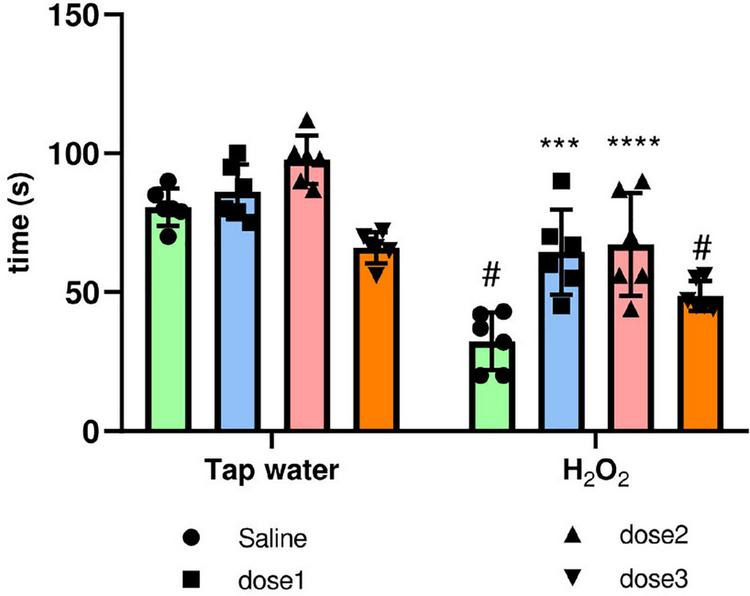
Effect of the interaction between the extract treatment and the exposure to H_2_O_2_ on motor coordination assessed by the rotarod test. The time spent on the cylinder was calculated. The results were presented as mean ± SD (*n* = 6). Data were analyzed by Two-ways ANOVA followed by Bonferroni’s *post-hoc* test; ***p ≤ 0.001, ****p ≤ 0.0001 compared to saline group in the same treatment conditions. (#) in comparison with the control group (group 1).

Oxidative stress causes motor coordination and impairment. The protective potential of *M. rotundifolia* aqueous extract against such deleterious effect was evaluated using the rotarod test. H_2_O_2_ intake significantly decreased the motor coordination in animals (Group 2) compared to control group (Group 1), (*p* ≤ 0.05). Animals treated with *M. rotundifolia* extract (62.5 and 125 mg/kg) spent significantly more time on the cylinder compared to H_2_O_2_ group (Group 2) (*p* ≤ 0.05). However, *M. rotundifolia* extract at the dose of 250 mg/kg did not show any significant effect, Figure 6.

##### 3.3.2.4. Y-maze test

**FIGURE 7:**
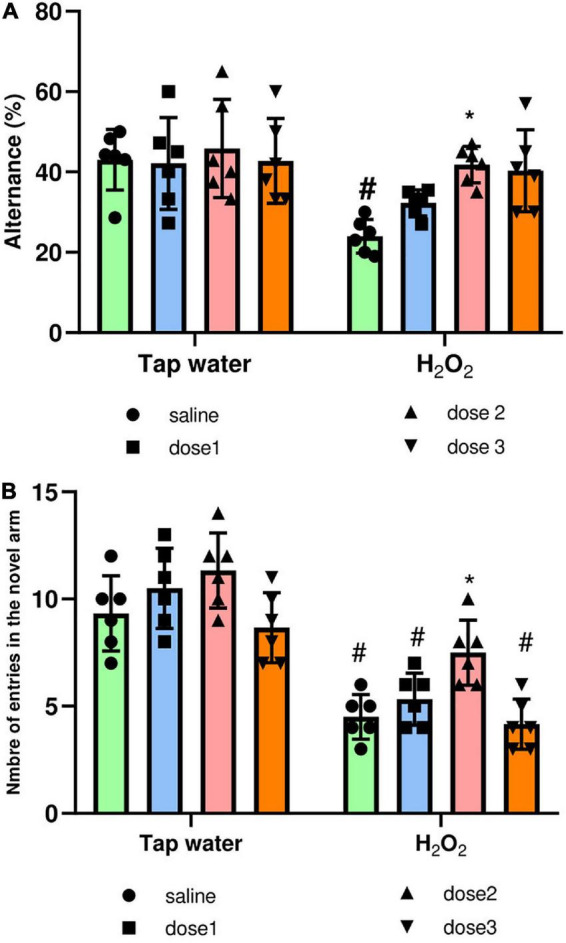
Effect of the interaction between the extract treatment and the exposure to H_2_O_2_ on short-term memory assessed by the Y-maze test. **(A)** Presents the percentage of alternance as an indicator of the spatial working memory. **(B)** Presents the number of entries in the novel arm as an indicator of spatial reference memory. The results were presented as mean ± SD (*n* = 6). Data were analyzed by Two-ways ANOVA followed by Bonferroni’s *post-hoc* test; *p ≤ 0.05 compared to saline group in the same treatment conditions. (#) in comparison with the control group (group 1).

The Y-maze test was used to assess the effect of oxidative stress on short-term memory and the protective effect of *M. rotundifolia* aqueous extract against memory impairment. The spatial working memory was evaluated by the measure of the spontaneous alternance, Figure 7A. Alternance was significantly (*p* ≤ 0.05) decreased in the H_2_O_2_ only administered group (group 2) compared to saline group (group 1). This effect was reversed by *M. rotundifolia extract* at 125 mg/kg, Figure 7A. The spatial reference memory was evaluated by calculating the number of entries in the novel arm, Figure 7B. H_2_O_2_ intake induced a significant reduction (*p* ≤ 0.05) in the number of entries in the novel arm in the groups (2, 4, and 8) in comparison with control group (group 1). This effect was reverted in 125 mg/kg extract treated group (Group 6), which had no significant difference (*p* > 0.99) with the control group (group 1).

##### 3.3.2.5. Tail flick test for hyperalgesia

**FIGURE 8:**
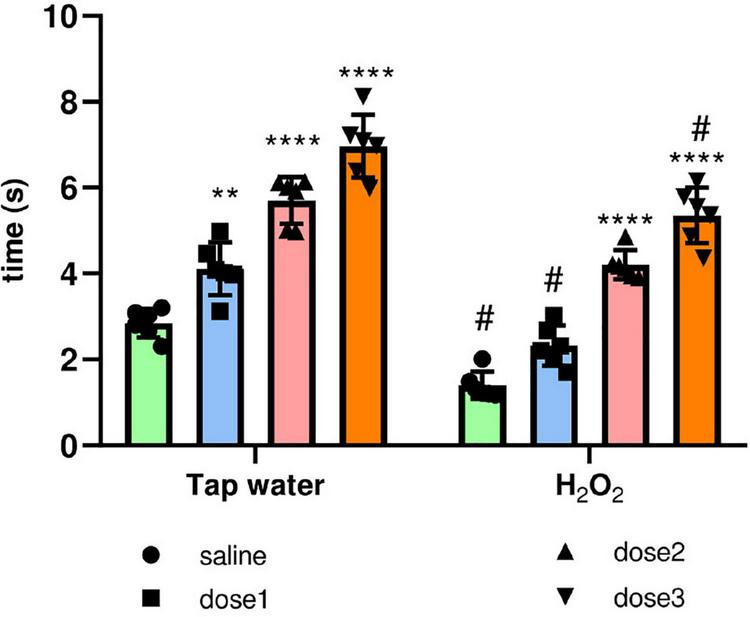
Effect of the interaction between the extract treatment and the exposure to H_2_O_2_ on hyperalgesia evaluated by the tail immersion test. The latency of the tail withdrawal reflex was measured. The results were presented as mean ± SD (*n* = 6). Data were analyzed by Two-ways ANOVA followed by Bonferroni’s *post-hoc* test; **p ≤ 0.01, ****p ≤ 0.0001 compared to saline group in the same treatment conditions. (#) in comparison with the control group (group 1).

Tail flick test was used to assess the effect of H_2_O_2_ exposure and treatment by the extract on pain perception. For this, the tail withdrawal reflex latency was recorded, Figure 8. H_2_O_2_ intake remarkably decreased (*p* ≤ 0.0001) the response time compared to the control group (Group 1). Pre-treatment with *M. rotundifolia* extract attenuated these effects at two tested doses (125 and 250 mg/kg). Noticeably, *M. rotundifolia* treated groups, at the three tested doses, had a significant (*p* ≤ 0.05) higher latency time independently from the oxidative stress, which demonstrates the analgesic effect of *M. rotundifolia*.

##### 3.3.2.6. Hepatic biomarkers concentrations

Hepatic enzymes were assayed as shown in Table 3. H_2_O_2_ intake markedly elevated the three tested biomarkers (ALT, AST, and total bilirubin) compared to the control group (Group 1). *M. rotundifolia* extract decreased the elevated levels of ALT and AST at the two tested doses 125 and 250 mg/kg and bilirubin at the three tested doses. Interestingly, *M. rotundifolia* at the dose of 250 mg/kg induced a significant mild elevation in the total bilirubin level (*p* ≤ 0.05) in comparison to control group (group 1). Additionally, there was no significant difference (*p* > 0.05) between the groups receiving this dose in “Tap water” (group 7) and those receiving H_2_O_2_ (group 8).

**TABLE 3:** Hepatic biomarkers results.

Groups	ALT		AST		Bilirubin	
	UI/L					
	Tap water	H_2_O_2_ (2%)	Tap water	H_2_O_2_ (2%)	Tap water	H_2_O_2_ (2%)
Saline	33.23 ± 2.35	48.56 ± 2.90^a^	48.85 ± 1.92	71.15 ± 2.82^a^	0.77 ± 0.09	35.42 ± 7.00^a^
Dose 1 (62.5 mg/kg)	31.78 ± 3.06	44.54 ± 4.49^a^	45.65 ± 1.99	69.16 ± 1.01^a^	1.91 ± 0.67	15.48 ± 2.62^a,b^
Dose 2 (125 mg/kg)	29.29 ± 2.43	35.858 ± 4.09^b^	47.30 ± 2.02	60.8 ± 1.81^a,b^	4.24 ± 0.66	9.21 ± 1.39^a,b^
Dose 3 (250 mg/kg)	29.97 ± 3.28	29.37 ± 2.39^b^	47.54 ± 1.54	49.25 ± 1.26^b^	6.735 ± 0.58*^a,b^	6.86 ± 0.52^a,b^

Results were presented as mean ± SD (*n* = 6). Data were analyzed by Two-ways ANOVA followed by Bonferroni’s *post-hoc* test.

^a, b^Significantly different from the Tap water group (Group 1) and H_2_O_2_ group (Group 2) at *p* ≤ 0.05.

## 4. Discussion

H_2_O_2_ is generated by several sources of oxidative stress. It’s considered as a signaling biological messenger due to its ability to diffuse freely into many types of tissues and membranes (Gammella et al., 2016). It damages important classes of biological macromolecules in cells directly by oxidizing lipids, proteins, and nucleic acids, which result in many diseases (Guesmi et al., 2018). The cells’ antioxidant defenses counteract H_2_O_2_ and ROS that have crossed the cell membrane. However, once they reach a threshold, cells eventually succumb to severe oxidative damage, leading to cell death (Law et al., 2014). Oxidative stress has been also linked to cognitive decline in several medical conditions, including aging, traumatic brain injury, and Alzheimer’s disease. In fact, most studies on cognitive impairment in Alzheimer’s disease link it to oxidative stress through decreased levels of antioxidant enzymes (Alzoubi et al., 2012).

In this context, the present study was conducted to evaluate the protective effect of the aqueous extract of *M. rotundifolia* leaves against H_2_O_2_ induced toxicity. H_2_O_2_ exposure significantly decreased the locomotion activity evaluated by the OF and Rotarod tests as it was shown with other substances inducing oxidative stress such as aspartame, clomipramine, and paraquat (Niveditha et al., 2017; Onaolapo et al., 2017; Othman et al., 2019). It also induced Anxiety-like symptoms defined by the number of entries and the time spent in the inner zone of the OF. In the Y-maze test, the spatial working memory and the spatial reference memory were represented by the spontaneous alternance and the number of entries in the novel arm, respectively, which was significantly decreased due to H_2_O_2_ exposure. As awaited, the plant treated groups showed less oxidative stress related behavior impairments especially at the doses of 125 mg/kg.

These findings may be attributed to the high content of neuroprotective compounds in *M. rotundifolia* extract. It was shown that kaempferol, identified in the extract, act as neuroprotective agent against rotenone-induced Parkinson’s disease model of rats by preventing the loss of tyrosine hydroxylase expression (Pan et al., 2020). In addition, its administration protected against Chlorpyrifos-induced oxidative stress and memory deficits in rats *via* GSK3β-Nrf2 signaling pathway and increased the activities of antioxidant enzymes and AChE (Hussein et al., 2018). Furthermore, the intracerebroventricular Micro-injection of Kaempferol reduced anxiety through the GABAergic mechanism by binding to specific sites of benzodiazepines (Zarei et al., 2021). However, in H_2_O_2_ conditions, it was observed that the group received a 250 mg/kg dose of *M. rotundifolia* extract had significantly less locomotor activity. It was thought that this was related to the toxicity or the sedative effect of the extract, but according to the literature, the plant is safe even at high doses and has no psychotropic effect (Bounihi, 2016). Further investigations should be carried out to explain these results.

Hyperalgesia was assessed using the tail immersion test. H_2_O_2_ exposure significantly decreased the latency of the tail reflex. Several studies have related hyperalgesia to oxidative stress and showed that it can be attenuated by antioxidant treatments (Mohajjel Nayebi et al., 2021). The extract treated groups, whether in “Tap water” or “H_2_O_2_” conditions showed dose-dependent responses. Tail withdrawal latency was maximal at 250 mg/kg suggesting an analgesic effect of *M. rotundifolia* extract. It was previously shown that at a dose of 600 mg/kg, *M. rotundifolia* extract had a greater effect than aspirin on acetic acid induced writhing in mice (Boussouf et al., 2017).

The liver is involved in the detoxification and elimination of xenobiotics, some of which are potentially toxic. This leads to oxidative damage of hepatocytes, reflected by the increase of hepatic enzymes. Additionally, liver plays a key role in the metabolism of orally and intra-peritoneally administered drugs (Turner et al., 2011), the two routes we chose for the administration of H_2_O_2_ and *M. rotundifolia* extract, respectively. It was even shown that impaired liver function was predisposing factor for encephalopathy and neurodegenerative diseases (Butterworth, 2013). Furthermore, in a cohort study of 1,581 older adults, elevated AST and ALT levels were associated with the diagnosis of Alzheimer’s disease, poor cognition, and reduced glucose metabolism in the brain (Nho et al., 2019). Thus, substances able to regulate liver biomarkers may be effective in preventing neurodegenerative diseases. The major antioxidant enzymes that can eliminate H_2_O_2_ are catalase, glutathione peroxidase and peroxiredoxins (Phaniendra et al., 2015). It was previously demonstrated that *M. rotundifolia* extract significantly improved the activities of these enzymes in the liver homogenate after carrageenan injection (Mohajjel Nayebi et al., 2021). Hepatic biomarkers (ALT, AST, and Bilirubin) were assayed as well in this work. The end products of some metabolic pathways such as uric acid, taurine, and bilirubin can have cytoprotective effects, but at the same time, they are harmful at high levels (Glazer, 1989). In the present study, we focused on the antioxidant effect of bilirubin. H_2_O_2_ exposure induced a significant hyperbilirubinemia accompanied by a significant increase in AST and ALT levels. *M. rotundifolia* extract significantly decreased the elevated levels in a dose-dependent manner. At a dose of 250 mg/kg, AST and ALT levels in H_2_O_2_ groups were comparable to those in “Tap water” groups.

In general, the characterized phytoconstituents have been already reported in prior works for their pharmacological properties. Quinic and chlorogenic acids have a wide range of biological activities such as antidiabetic, DNA protective, neuroprotective, hepatoprotective, and antifungal effects (Ma and Ma, 2015; Zuo et al., 2015). Caffeoyl shikimic acid showed an anti-hyperuricemia effect and ameliorated kidney injury in mice (Zhang et al., 2021). Kaempferol and its derivatives are flavonols with promising bioactivities including anti-cancer, antiviral, antimicrobial, and neuroprotective properties (Bangar et al., 2022). Additionally, all these compounds have been shown to possess an important antioxidant activity (Sato et al., 2011; Uzun et al., 2017; Riahi et al., 2019), which may explain the strong antioxidant potential of *M. rotundifolia* aqueous extract in DPPH and ABTS assays, as it was previously confirmed (Fatiha et al., 2015; Siham et al., 2019).

Noteworthy, when bilirubin acts as an antioxidant, it can protect the cells against up to 10,000 times higher concentrations of H_2_O_2_ (Doré et al., 1999). In the current work, administration of *M. rotundifolia* extract resulted in a mild elevation in bilirubin level. This may be due to the richness of *M. rotundifolia* in flavonoids as it was previously demonstrated with isolated flavonoids from of the Milk thistle, *Silybum marianum* L., induced elevated bilirubin concentrations (Šuk et al., 2019). Patients with Gilbert’s syndrome, which is characterized by mild elevated bilirubin, have been shown to have a significant lower level of oxidative stress without experiencing symptoms of toxicity related to elevated bilirubin levels (Maruhashi et al., 2012). Furthermore, it was found that the induction of mild hyperbilirubinemia may have an important therapeutic potential specially to prevent diseases related to oxidative stress such as cardiovascular disease, type 2 diabetes, metabolic syndrome, and neurodegenerative disorders (Vitek et al., 2019). These findings suggest that the *M. rotundifolia* leaves have an important antioxidant potential, and their phytoconstituents may be good candidates to develop new drugs for oxidative stress comorbidities.

Substantial differences were found at the chemical composition of *M. rotundifolia* from the Moroccan flora compared to those from other countries. In the current work, kaempferol glucuronide (accounted for 85%) dominated the extract along with other minor compounds, among them quinic acid, chlorogenic acid, caffeoyl shikimic acid, and kaempferol rutinoside. Noteworthy, rosmarinic acid was detected at the extract as a minor compound; however, it was among the major compounds from the Algerian and Tunisian species (Fatiha et al., 2015; Siham et al., 2019). While eriocitrin was predominant in Saudi Arabian flora (Alharbi et al., 2021). These variations may be explained by several factors such as geographical distribution, temperature, water availability and soil composition (Kabtni et al., 2020).

## 5. Conclusion

*Mentha rotundifolia* L. is traditionally used for its analgesic, antispasmodic, and anti-inflammatory properties. The current study is the first of its kind carried out for this plant in terms of its neuroprotective potential in an *in vivo* model. *M. rotundifolia* extract furnished *in vitro* antioxidant activities, reduced the effects of H_2_O_2_-induced oxidative stress, and attenuated the associated behavioral changes. These activities may explain its traditional uses in health and nutrition supplements and promotes its valorization as a nutraceutical. However, further analysis should be performed for better understanding of its neuroprotective effect pathway, isolate its individual components, and determine its therapeutic dose, onset and duration of action.

## Data availability statement

The raw data supporting the conclusions of this article will be made available by the authors, without undue reservation.

## Ethics statement

All animal procedures were conducted in accordance with international ethical standards (European Union Directive 2010/63/EU) legislation and ARRIVE (Animal Research Reporting of In Vivo Experiments) guidelines.

## Author contributions

KB, NB, and MS designed the study, analyzed the data, and contributed to the writing of the manuscript. MT and KT supervised and provided critical feedback that shaped the final version of the manuscript. All authors contributed to the article and approved the submitted version.
