# Supplementary figures and images for: Mentha rotundifolia (L.) Huds. aqueous extract attenuates H2O2 induced oxidative stress and neurotoxicity
Source: Front Neurosci. 2023 Mar 9;17:1121029. doi: 10.3389/fnins.2023.1121029 (PMC10035595; doi:10.3389/fnins.2023.1121029)

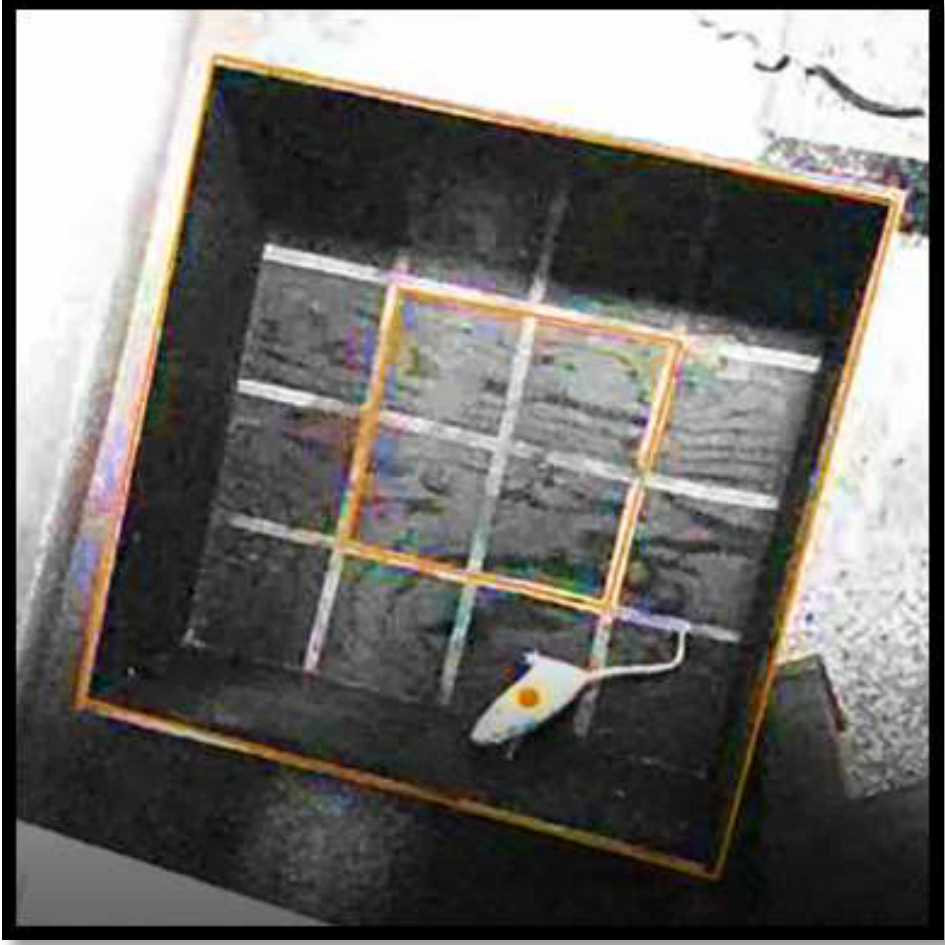

Supplement: Supplementary file 1 [file Image_1.TIF]

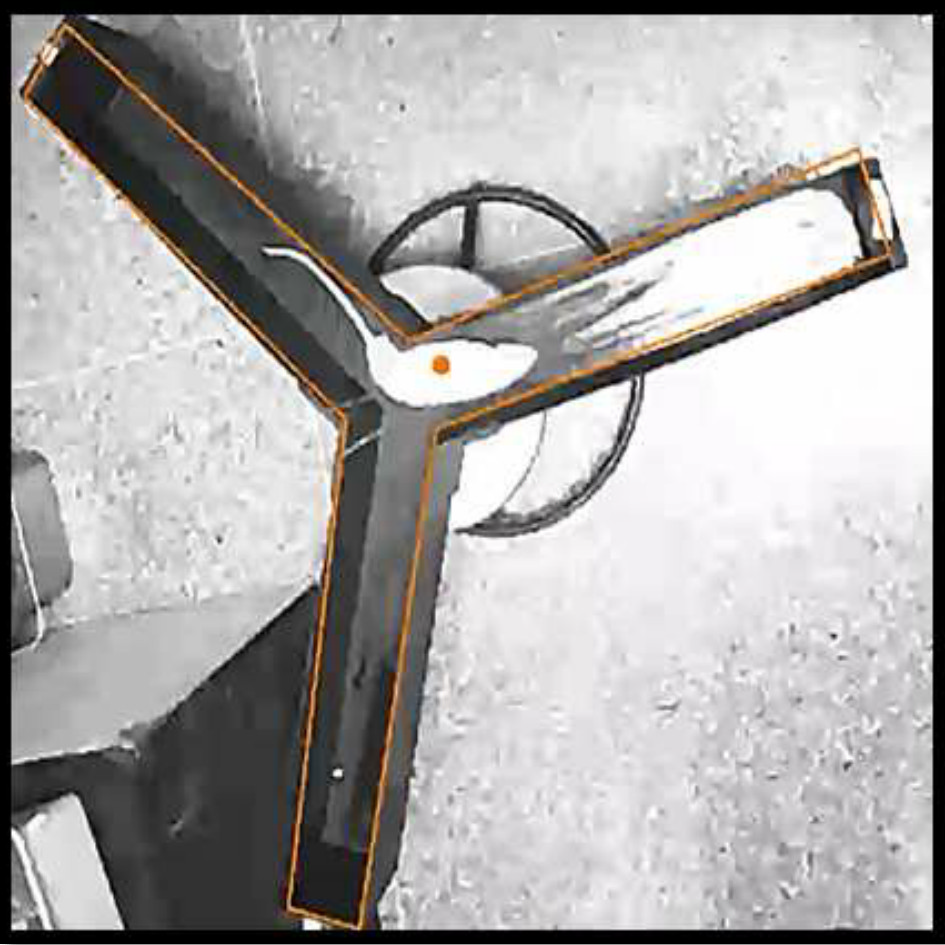

Supplement: Supplementary file 2 [file Image_2.TIF]

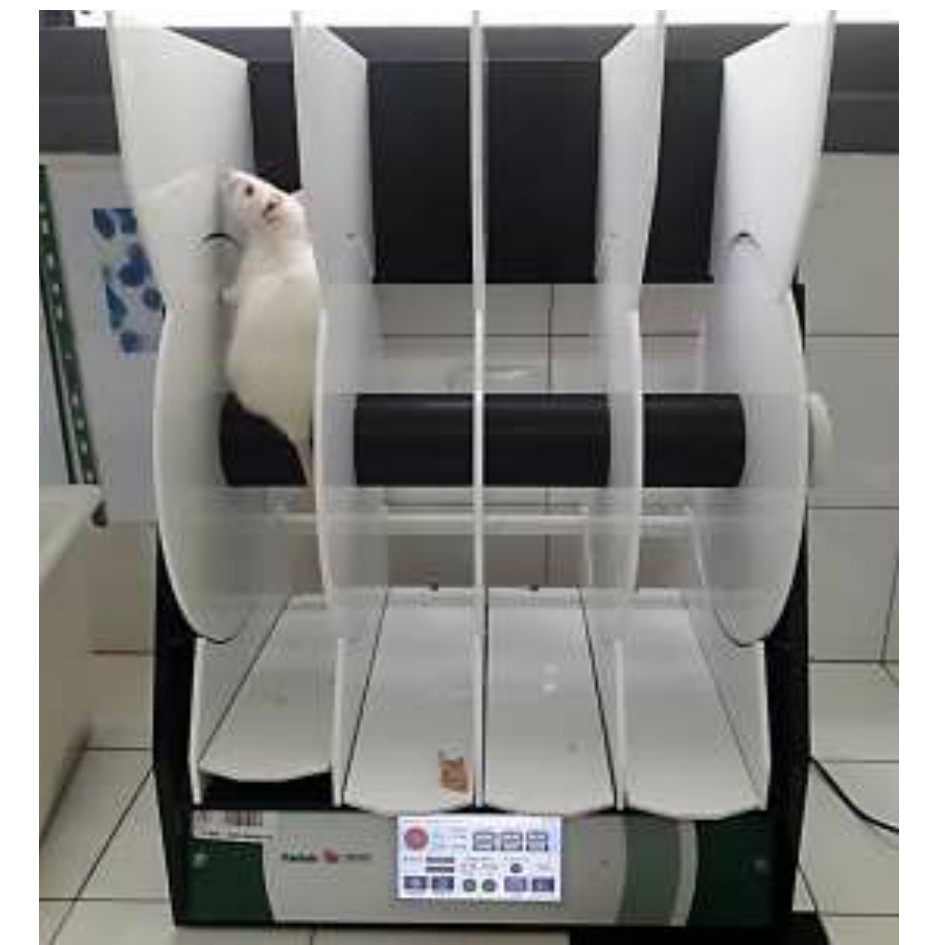

Supplement: Supplementary file 3 [file Image_3.TIF]

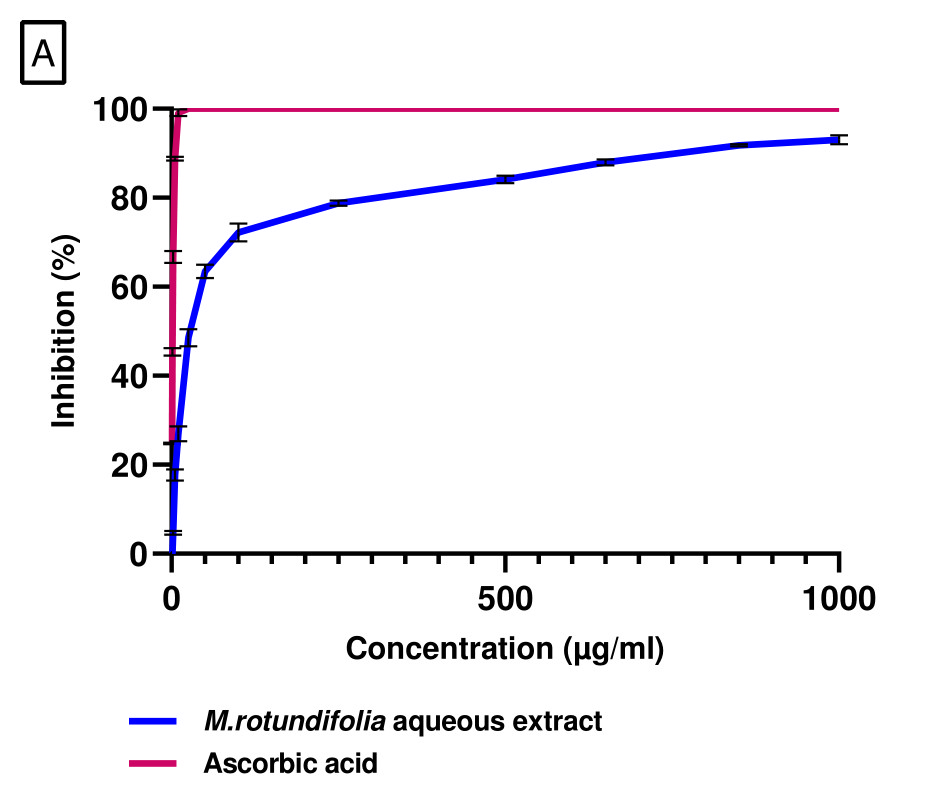

Supplement: Supplementary file 4 [file Image_4.TIF]

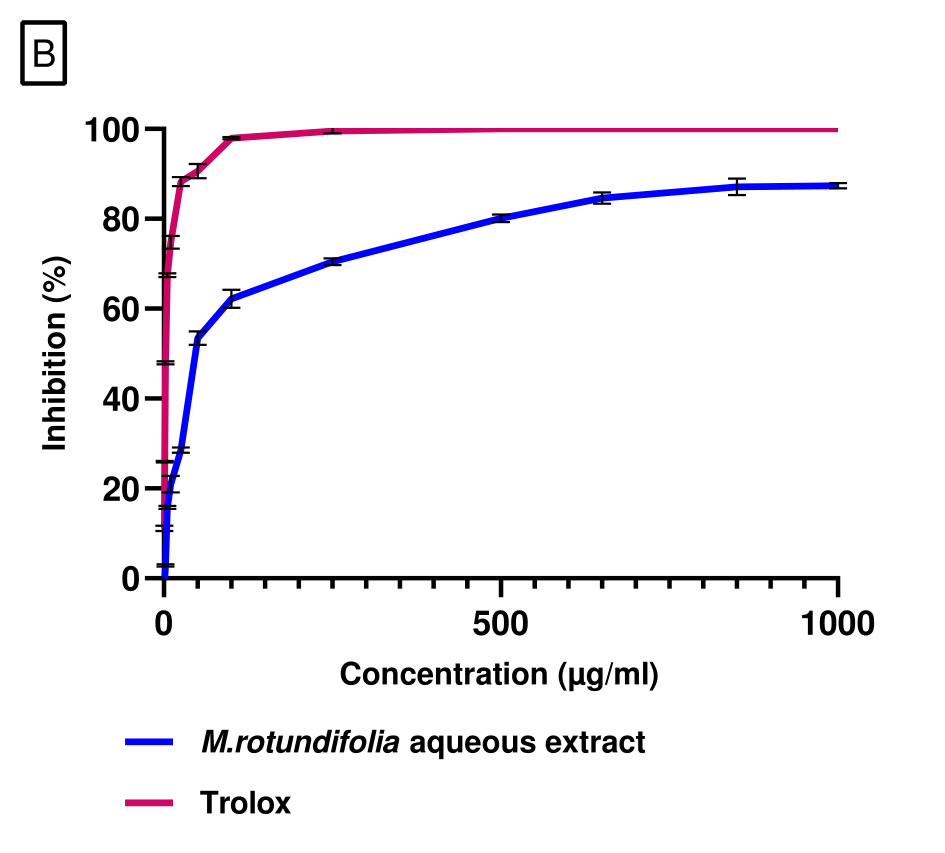

Supplement: Supplementary file 5 [file Image_5.TIF]
